# Optimization of Sensors to be Used in a Voltammetric Electronic Tongue Based on Clustering Metrics

**DOI:** 10.3390/s20174798

**Published:** 2020-08-25

**Authors:** Munmi Sarma, Noelia Romero, Xavier Cetó, Manel del Valle

**Affiliations:** Sensors and Biosensors Group, Department of Chemistry, Universitat Autònoma de Barcelona, 08193 Barcelona, Spain; Munmi.Sarma@uab.cat (M.S.); noelia.romeropr@e-campus.uab.cat (N.R.); xavier.ceto@uab.cat (X.C.)

**Keywords:** electronic tongue, voltammetric sensors, principal component analysis, artificial neural networks, discrete wavelet transform

## Abstract

Herein we investigate the usage of principal component analysis (PCA) and canonical variate analysis (CVA), in combination with the F factor clustering metric, for the a priori tailored selection of the optimal sensor array for a given electronic tongue (ET) application. The former allows us to visually compare the performance of the different sensors, while the latter allows us to numerically assess the impact that the inclusion/removal of the different sensors has on the discrimination ability of the ET. The proposed methodology is based on the measurement of a pure stock solution of each of the compounds under study, and the posterior analysis by PCA/CVA with stepwise iterative removal of the sensors that demote the clustering when retained as part of the array. To illustrate and assess the potential of such an approach, the quantification of paracetamol, ascorbic acid, and uric acid mixtures were chosen as the study case. Initially, an array of eight different electrodes was considered, from which an optimal array of four sensors was derived to build the quantitative ANN model. Finally, the performance of the optimized ET was benchmarked against the results previously reported for the analysis of the same mixtures, showing improved performance.

## 1. Introduction

Electronic tongues (ETs) are analytical systems based on the combination of an array of sensors with low-selectivity and/or cross-response features in order to obtain some added value in the generation of analytical information. These are coupled with advanced chemometric tools that allow the interpretation and extraction of meaningful data from the complex readings [1,2]. Thus, the selection of the sensor array that will comprise the ET is a key step that will highly influence the performance of the system [3].

Despite its importance, most of the papers dealing with ETs focus on the developed application itself or the data treatment stage, but very few report on the choice of the sensors. The challenge here arises on the a priori selection of the best combination of sensors that can carry out the desired qualitative or quantitative task given the difficulty to assess the cross-reactivity shown between them and the impact that this will have in the final model. In this direction, one common approach that has been taken in the case of potentiometric sensors is the inclusion of at least one or two ion-selective sensors (ISEs) towards the compounds of interest plus some generic ones; however, this does not guarantee that the optimal array is selected [1]. Another approach would be the complete sensor characterization, which consists in carrying out the multi-analyte calibration, from which the surface response plots are built and the binary selectivity coefficients are calculated (e.g., Reference [4]). However, this is a tedious task even for the two-analyte case, and it does become not feasible for more complex mixtures as it may require hundreds of samples. Furthermore, such a task becomes even more complex when, e.g., voltammetric sensors are used instead of potentiometric ones due to the higher complexity and dimensionality of its response. Alternatively, another approach that has been employed is the use of feature selection methods upon measurement of all the samples, and to carry out the a posteriori removal of the variables and/or sensors that do contribute less to the classification or quantification task (e.g., usage of genetic algorithms or other pruning methods [5]). Although this approach does probably provide the best outcome possible, it is also more tedious and requires instruments capable of simultaneously measuring a large number of channels.

In the same direction, we had also reported previously on the usage of the autocorrelation between the signals of the different sensors that form the sensor array as an objective criterion for the selection or removal of redundant sensors in voltammetric arrays [6,7]. Although it provides a measure of sensors’ response redundancy, it is purely based on the signal, but not on the cross-reactivity towards the analytes of interest. More recently, principal component analysis (PCA) taking the transposed data matrix has also been suggested as a guiding method to select the best sensing units to compose the array of an ET [8]. However, the authors themselves conclude that further experiments are required to confirm the potential of the method.

Therefore, the development of a simple methodology that allows the a priori selection of the optimal sensor array to carry out a specific application is of utmost interest. In this direction, herein we propose the usage of PCA in combination with some clustering metrics as a tool to carry out such selection. The former allows us to visually compare the performance of the different sensors, while the latter allows us to numerically assess the impact that the inclusion/removal of the different sensors does have in the discrimination ability of the ET towards the compounds of interest.

In order to demonstrate and illustrate such a procedure, the simultaneous quantification of paracetamol (PA), ascorbic acid (AA), and uric acid (UA) mixtures was chosen as the study case. These mixtures correspond to a common case in the pharmaceutical field where the determination of paracetamol in the presence of ascorbic acid is attempted. The latter is usually present as an excipient, whereas the inclusion of uric acid is motivated as some studies suggest that ascorbic acid intake is related to uric acid concentration in serum [9,10]. This particular case was chosen as this mixture has already been previously analyzed in our laboratories employing different sensors arrays, thus providing us with guidance on which performance could be expected and whether previous results could be improved or not by tailoring the electrode choice to particular cases.

In this direction, the present work aims to demonstrate the advantages derived from the tailored selection of the sensor array for each ET developed application. Upon measurement of stock solutions of the different active pharmaceutical ingredients (APIs), those were transformed by using PCA/CVA, which in combination with the F factor metric allowed the selection of the different sensors. Next, a quantitative model was built by means of artificial neural networks (ANNs) to achieve the simultaneous determination of the three APIs, the performance of which was benchmarked against previously reported ETs.

## 2. Materials and Methods

### 2.1. Reagents and Apparatus

All reagents were of analytical reagent grade and were used as received without any further purification. All the buffer solutions were prepared in ultrapure water (18.2 MΩ·cm) purified by a MilliQ System (Millipore, Billerica, MA, USA). Potassium hydrogenphosphate, potassium dihydrogenphosphate and sodium chloride, which were used for the preparation of the phosphate buffer, were purchased from Merck (Darmstadt, Germany).

The active pharmaceutical ingredients (APIs), paracetamol and uric acid, were purchased from Sigma-Aldrich (St. Louis, MO, USA), whereas ascorbic acid was purchased from Panreac Química SLU (Barcelona, Spain). For the modification and preparation of the electrodes, we used nanoparticles of bismuth (III) oxide, titanium (IV) oxide, zinc (II) oxide and tin (IV) oxide, plus cobalt (II) phthalocyanine and polypyrrole purchased from Sigma-Aldrich (St. Louis, MO, USA), while Prussian blue was obtained from Acros Organics (Geel, Belgium). Graphite powder (particle size < 50 µm) used for the construction of the electrodes was purchased from BDH Laboratory Supplies (Poole, UK), and Epotek H77 resin and the corresponding hardener were obtained from Epoxy Technologies (Billerica, MA, USA).

All the electrochemical measurements were carried out in a PGSTAT 30 Autolab potentiostat (EcoChemie, The Netherlands) with GPES 4.7 version software (EcoChemie). Voltammetric measurements were conducted using a conventional three-electrode cell configuration where a combined electrode (Crison 5261, Barcelona, Spain), made up of a metallic platinum wire and an Ag/AgCl electrode was used as both the auxiliary and reference electrode.

### 2.2. Sensor Array

An array of seven different modified graphite epoxy composite (GEC) electrodes was initially prepared to be evaluated as the working electrodes that will form the ET [11]. Briefly, for the construction of the working electrodes, first of all, a shaped copper disc was soldered to an electrical connector, and then introduced into a 6 mm internal diameter PVC tube, resulting in a cylindrical cavity. A paste was then made by mixing 15% of graphite powder, 2% of the specific modifier, and the epoxy resin and the hardener (in the ratio 20:3 w/w). Next, this paste is loaded into the cavity of the PVC tube and cured at 80 °C for 3 days. Afterwards, using emery papers of decreasing grain size, electrode surfaces were polished until a flat shiny surface appeared. One of the main advantages of such electrodes is that re-polishing of the surface using emery paper allows the regeneration of the electrode, recovering any loss of their response.

In this manner, seven different GEC electrodes, each of them modified with cobalt (II) phthalocyanine (CoPc), polypyrrole (PPy), Prussian blue (PB), oxide nanoparticles of bismuth (Bi_2_O_3_), titanium (TiO_2_), zinc (ZnO) and tin (SnO_2_), were prepared. Additionally, a Pt disc electrode was also prepared and used to complete the eight-sensor array. The Pt disc electrode was constructed by soldering a Pt wire (99.95% purity, 1 mm diameter) to an electrical connector and then introducing the connector into a PVC tube. The wire was then coated in epoxy resin (exposing only the wire cross-section) and cured at 80 °C for 3 days.

Such modifiers were selected taking into account previous studies with ETs. Prussian blue is well-known to be an electron mediator in the development of many biosensors, which has also been used in the development of ETs [12,13,14]. The usage of nanoparticles has emerged as an alternative to the respective bulk metals given its higher surface/mass ratio and improved electrochemical properties [13,15,16]. Similarly, conducting polymers such as polypyrrole have electrocatalytic and antifouling properties [17,18,19], while phthalocyanines are reported to be efficient electrocatalysts in the determination of many important inorganic, organic, or biological compounds [20,21]. Lastly, the use of bare metal electrodes corresponds to one of the more common choices for the development of voltammetric ETs [22,23,24].

### 2.3. Samples Preparation and Voltammetric Measurements

All APIs stock solutions were prepared in 0.05 M phosphate buffer at pH 7.0 with 0.1 M KCl as saline background/supporting electrolyte. For the optimization of the sensor array, 250 μM stock solution of each of the APIs were measured separately in order to investigate their electrochemical behavior with the different sensors. Upon selection of the optimal sensor array, calibration curves for the APIs were built and their analytical response was further characterized in terms of linearity, sensitivity, limit of detection (LOD), reproducibility, etc. To this aim, solutions of increasing concentration of each API were prepared from the stock solutions in phosphate buffer and measured under the below conditions.

For the simultaneous analysis of APIs mixtures, a set of samples consisting of mixtures of the three compounds were prepared. Samples were divided into two subsets: the training subset based on a tilted 3^3^ factorial design (27 samples), which was used to build the quantitative model [25], and the testing subset with samples randomly distributed along the experimental domain (11 samples) that was used to assess the actual performance of the built models.

The electrochemical behavior of all the APIs and their mixtures was assessed by recording a complete cyclic voltammogram between −0.7 V and +1.2 V vs. Ag/AgCl with a step potential of 10 mV and a scan rate of 100 mV·s^−1^, without the application of any pre-conditioning potential or accumulation time. Furthermore, to avoid any fouling effect or drifts during the measurements, a blank measurement in phosphate buffer was carried out after each measurement.

These conditions were used for all the experiments, except during initial experiments for the selection of the sensor array, that in order to ensure that the potential window was wide enough to see any possible peak, voltammetric measurements were carried out in the range −1.5 V to +1.5 V, but keeping all the other conditions unaltered. Similarly, as a 6-channel multipotentiostat was employed, final measurements were carried simultaneously, but some of the initial measurements for sensor selection had to be carried sequentially in groups.

### 2.4. Chemometric Analysis

Data analysis was carried out in Matlab 7.1 (MathWorks, Natick, MA, USA) by specific routines developed by the authors using the Statistics, Wavelet, and Neural network toolboxes [26,27,28]. Final representation and analysis of the data were done with the aid of Sigmaplot (Systat Software Inc., San Jose, CA, USA).

Recorded voltammograms were first compressed with discrete wavelet transform (DWT), which allowed us to decrease the dimensionality of the data while preserving the relevant information [26]. Next, PCA and canonical variate analysis (CVA) were used for the qualitative analysis of the data and a tailored selection of the ET array. Finally, ANNs were used to build the simultaneous quantitation models of the ternary mixtures.

For the selection of the optimal sensor array, data was submitted to PCA and CVA, and the clustering observed was evaluated by means of the F factor [29,30,31]. The F factor is defined as the ratio of variances between different clusters and the sum of internal variance in all clusters (Equation (1)). It can be used as a standard procedure to measure the capability of a particular sensor to discriminate between different classes of samples in ET applications. Since the F factor compares the variability between classes to variation within classes, with increasing F values the discrimination between different classes becomes easier.

(1) $$F=\frac{\frac{\sum_{i=1}^{k}n_{i}{\left(\bar{z_{i}}-\overset{=}{z}\right)}^{2}}{k-1}}{\frac{\sum_{i=1}^{k}\sum_{j=1}^{n_{i}}{\left(z_{ji}-\bar{z_{i}}\right)}^{2}}{\sum_{i=1}^{k}n_{i}-k}}$$

where *k* is the number of classes, *i* the number of following class, *j* the following number of the sample in *i*-th class, *n_i_* the number of samples in *i*-th class, and *z_ji_* the sensor response for *j*-th sample in *i*-th class, and $\bar{z_{i}}$ and $\overset{=}{z}\;$ are the mean value of a sensor response in a particular class of samples and the mean value of sensor response for all samples, respectively. These can be defined as:

(2) $$\bar{z_{i}}=\frac{\sum_{j=1}^{n_{i}}z_{ji}}{n_{i}}$$

(3) $$\overset{=}{z}=\frac{\sum_{i=1}^{k}\sum_{j=1}^{n_{i}}z_{ji}}{\sum_{i=1}^{k}n_{i}}$$

## 3. Results and Discussion

### 3.1. Selection of Sensors

**Figure 1:**
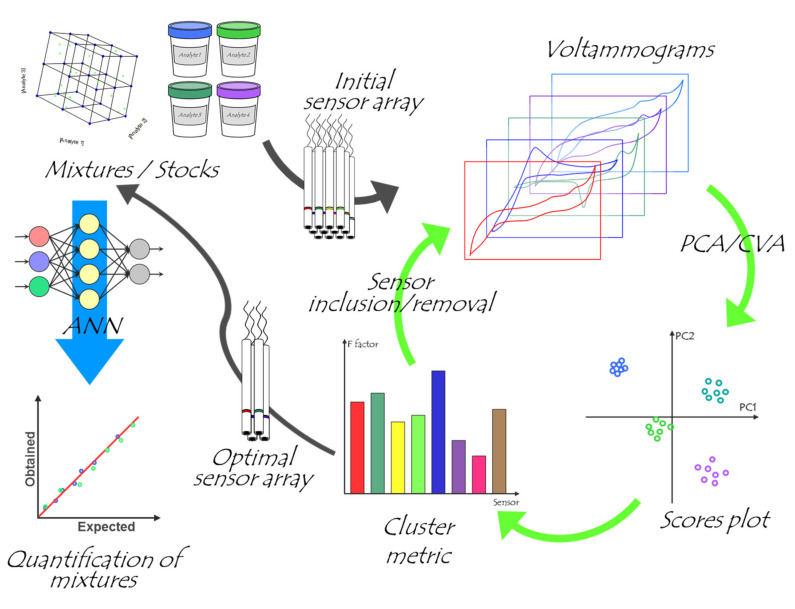
Schematic representation of the methodology followed for the a priori selection of the optimal sensor array. Briefly, stock solutions of each of the analytes are measured with all the considered sensors, obtaining a voltammogram for each of them. Next, those are submitted to PCA/CVA, and the clustering is evaluated by means of the F factor. This is repeated, leaving out of the analysis each of the sensors of the array (one at a time), and the one that leads to the higher improvement is removed. The whole process is repeated until a decrease in the F factor is observed after discarding one of the sensors. Finally, with the selected sensor array, the quantitative application is carried out.

The aim of this work is to demonstrate the advantages derived from the tailored selection of the optimal sensor array for each case when developing ET applications, and to assess whether this can be done a priori taking only single measurements of the stock of each of the compounds that we aim to analyze (Figure 1).

To this aim, we took as a study case the analysis of three different APIs (PA, AA and UA), and attempted to choose the most appropriate electrodes for their electrochemical analysis. When choosing the optimal electrodes, it has to be kept in mind how important the cross-responses of the different electrodes towards the analytes of interest and among them are in ET applications. That is, each electrode shows a differentiated response between each of the analytes, and the different sensors show a differentiated response between them, so that they jointly allow us to differentiate the different compounds [3].

**Figure 2:**
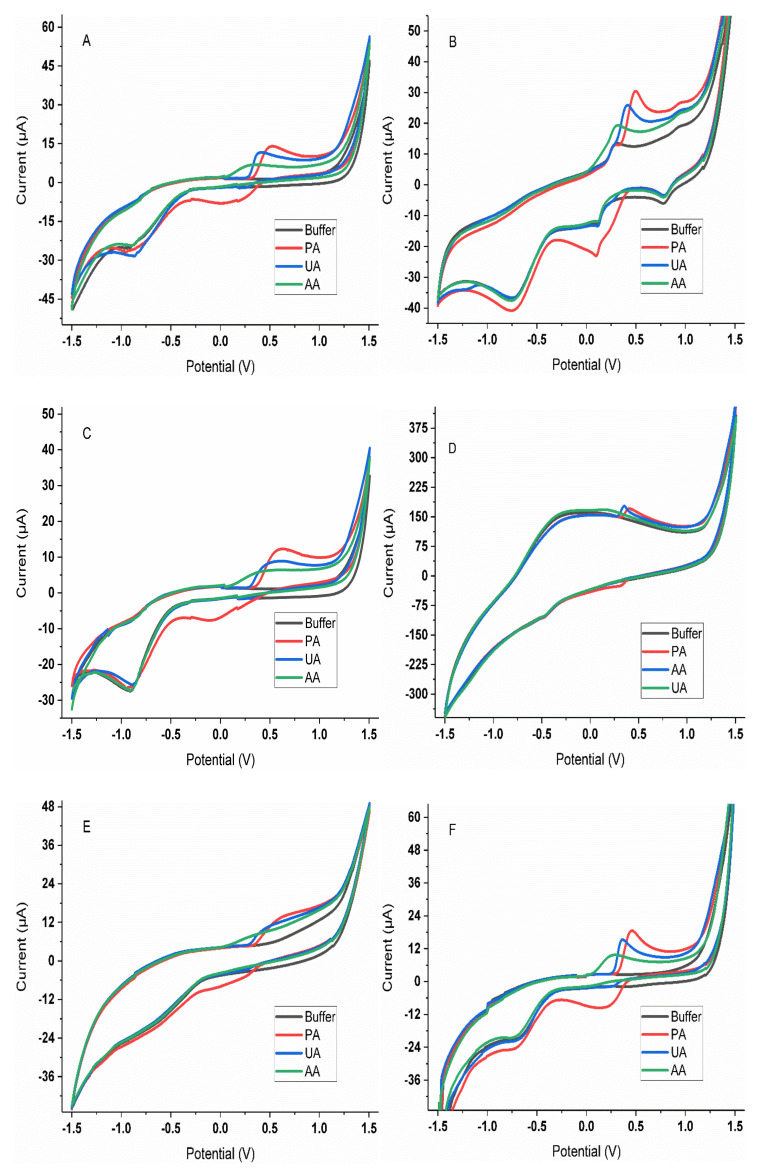

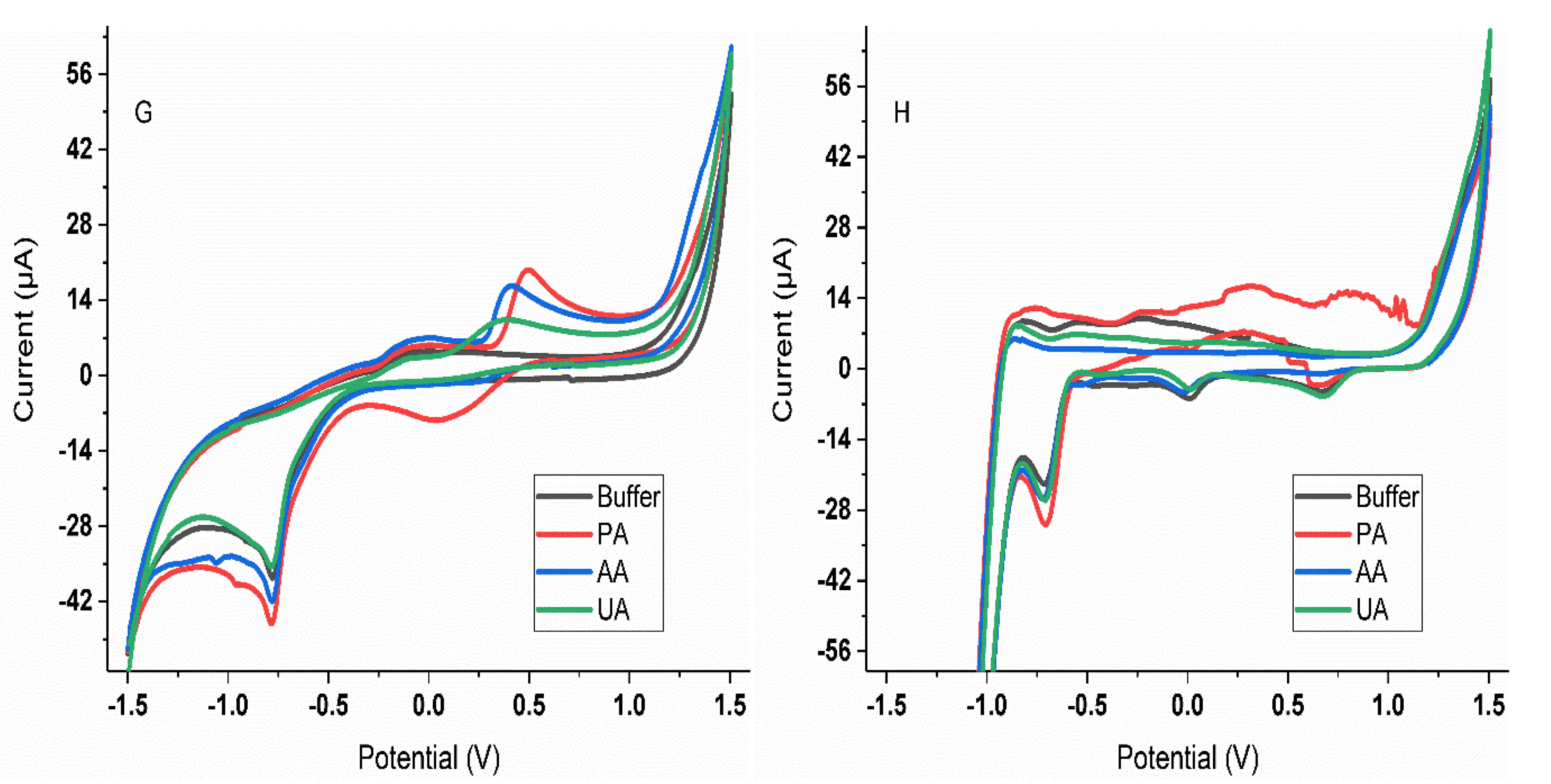
Voltammograms obtained for the three APIs (250 µM in phosphate buffer) using the GECs modified with (**A**) SnO_2_, (**B**) Prussian Blue, (**C**) ZnO, (**D**) PPy, (**E**) CoPc, (**F**) TiO_2_ and (**G**) Bi_2_O_3_, and (**H**) the metallic Pt electrode.

In this direction, the first step was to assess the voltammetric responses of each of the electrodes towards the individual compounds. For doing so, five replicate samples of the 250 µM solutions of each of the APIs were prepared, and the voltammetric measurements were carried as described in Section 2.3. An extract of the responses obtained for the different electrodes is shown in Figure 2. As it can be seen, the voltammetric profiles of each of the sensors are found to be different, with all of them showing distinct responses for each of the compounds.

**Figure 3:**
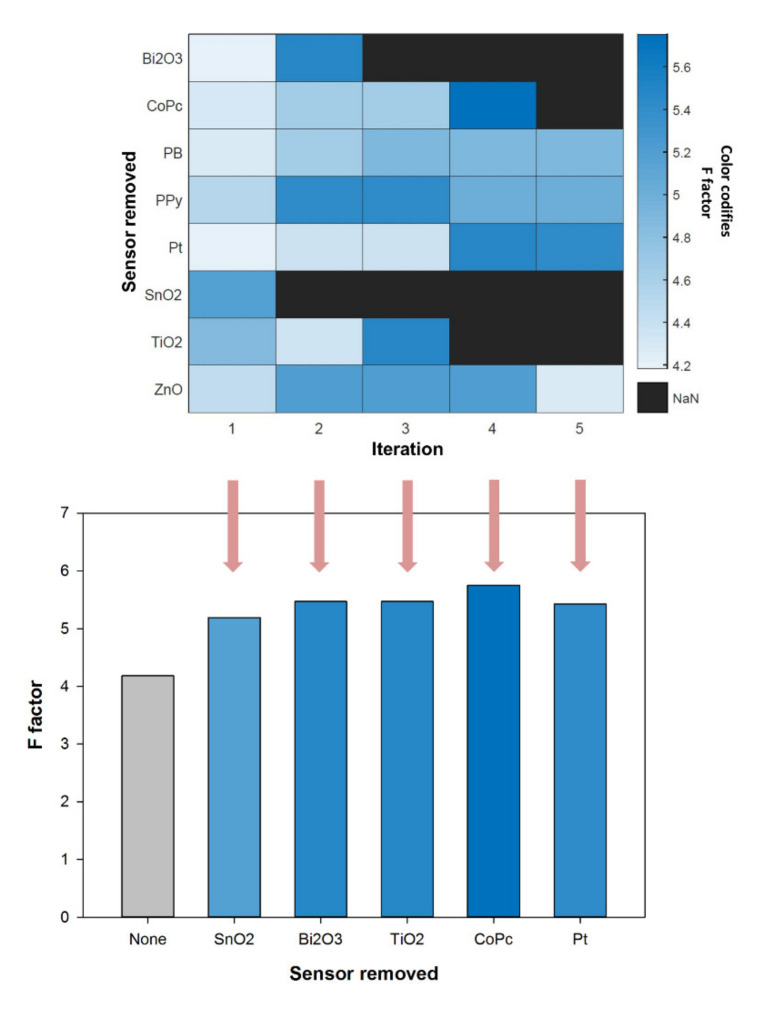
(Top) Color map of the variation of the F values after iterative exclusion of the different electrodes; the Y-axis shows the sensor being left out for the calculation of the F factor and the X-axis shows the successive iterations for the selection of the less significant sensor. (Bottom) Bar plot of the changes of the F values after exclusion of the sensor that leads to the biggest F value at each iteration. In both cases, the color of the plot codifies the F factor values as per the color bar. Iteration 5 does not produce any further improvement, and then the process is stopped.

To objectively carry out the selection of the final sensor array for the quantitative task, the next step was the compression of the data with DWT and its analysis by means of PCA and CVA. Firstly, the 2D score plots were obtained, and from those the F factor was calculated. This process was done with the 8-sensor array and repeated by removing one sensor at a time, similar to what could be considered a leave-one-out process, but from the sensors’ side (Figure 1). Next, the F values for each of the iterations were compared, and the sensor that when excluded led to the higher increase of the F index was then discarded. This process was repeated, reducing one-by-one the selected sensors, until the F value decayed (Figure 3).

In this manner, from the eight sensors initially considered, the first one that was discarded was the one modified with SnO_2_ nanoparticles, which led to a significant improvement in the clustering (the F value increased from 4.19 to 5.19). As stated, this process was repeated, taking the remaining seven sensors as reference for the F value, and leaving out again one sensor at a time for the calculations; the Bi_2_O_3_ nanoparticle modified sensor was discarded this time. In the next iteration, TiO_2_ modifier was discarded as no significant increase/decrease in the F value was observed. Thus, it was considered that its inclusion/exclusion does not demote the performance of the system. Lastly, the cobalt(II) phtalocyanine modified sensor was also discarded, whereas in the next iteration no further sensors were removed, as even the exclusion of the one with a larger F value implied a decrease of this parameter. In this manner, the selected sensor array was formed by four electrodes: a metallic Pt sensor and GECs modified with ZnO nanoparticles, Prussian blue, and polypyrrole, and the maximum F value achieved was 5.75.

**Figure 4:**
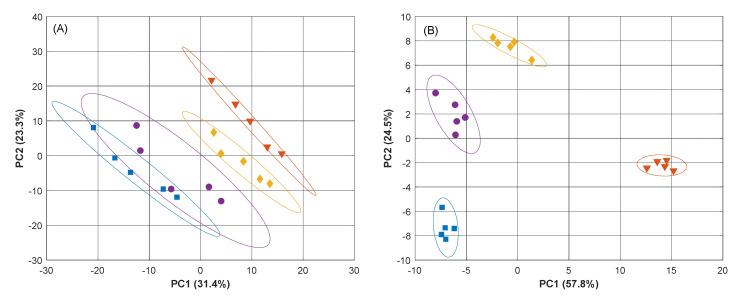
Score plot obtained from the DWT-PCA of five replicas of individual APIs using (**A**) the eight-sensor array or (**B**) the selected four-sensor array. (■) Buffer, (▼) paracetamol, (●) ascorbic acid and (♦) uric acid. Ellipses plotted correspond to 95% confidence limits for each of the clusters.

The resulting PCA score plot obtained with the reduced sensor array is shown in Figure 4B, with an accumulated explained variance of ca. 82.3 %; a large value that reflects how most of the variance contained in the original data is now summarized with only these two coordinates (PCs). More importantly, we can observe how clear clusters are obtained for each of the compounds and how easily these can be distinguished from each other. Hence, based on the voltammetric profiles and the PCA score plot, we can say that the initial selection of sensors based on the F factor calculation seems to be a satisfactory step towards the electrochemical quantification of the three APIs. Figure 4A, on the other hand, shows the departing point with the eight-sensors array, and where the clustering is just preliminarily worked out. As an additional comment, it is also evident how the optimization of the array is also able to amend the drift content incorporated in the original set of sensors.

Lastly, the analysis of the loadings biplot is not very significant in this case as what we aim to evaluate is not only which features are more relevant, but which improve more the discrimination capabilities of the ET. However, it is quite well known that since PCA is focusing only on the data variance, external factors, e.g., drift on the sensors response, can dominate it. Therefore, such information cannot be only obtained from the scores and loadings plot, but from the F factor. Moreover, the large number of variables registered when voltammetric sensors are used (hundreds for each of the sensors) makes the interpretation of the loadings plot difficult/cumbersome. However, a numerical inspection of the loadings contribution from the magnitude of its vector allowed us to confirm that in both cases all the sensors contribute significantly to the obtained scores plot, with most of the loadings very close to the correlation circle: ca. 81% of them with a magnitude as big as 0.6 times the one of the loading with the highest contribution for (A), and 72% for (B). In terms of sensors, the number of variables for each of them that contribute to those percentages are 15 to 30% for (B), and 2.2 to 17% for (A) (data not shown).

### 3.2. Characterization of the Analytical Response

Upon selection of the reduced sensor array, the next step was the characterization of the voltammetric responses of each of the electrodes towards the individual APIs. On the one side, we wanted to confirm that, actually, different sensitivity was shown by the different sensors, as well as assess its linear range to restrict the experimental domain for the quantitative experiment. On the other side, we wanted to also assess sensors’ repeatability, as this is a critical parameter when working with ETs, which require performing a large number of consecutive measurements.

#### 3.2.1. Calibration Curves

Prior to building the quantitative model for the mixtures’ analysis, the linear range and sensitivity of the different electrodes were evaluated. Individual calibration curves were built for each of the selected four sensors towards each of the APIs. To do so, stocks of increasing concentration within 0 to 500 µM for paracetamol and uric acid, and 0 to 2000 µM for ascorbic acid were prepared and measured as described in Section 2.3. From the recorded voltammograms, the maximum peak height was taken and plotted against its concentration (data not shown). From those, the fitted regression equations are summarized in Table 1. As represented by the large value of the coefficient of determination (R^2^), it can be concluded that good linearity was obtained within the above ranges for all the APIs and sensors. Hence, those same ranges were selected for the final quantitative model. Moreover, we can confirm how different sensitivities (calibration slopes) are obtained for each of the sensors and compounds, a situation that we aimed for with the selection of the sensor array (i.e., to avoid having redundant sensors or sensors that do not contribute to the discrimination of the different compounds).

#### 3.2.2. Stability Measurements

Similarly, since building the quantitative model requires a considerable number of consecutive measurements with the sensor array, it is important to assess whether the repeatability within consecutive measurements for all the sensors is good enough. In this direction, a sample containing 100 µM of each of the APIs was prepared, and the performance of the sensor was evaluated by calculating the relative standard deviation (RSD) obtained after 18 consecutive measurements without changing the sample, equipment, and analyst.

Before the sample measurement, a blank buffer measurement was taken, repeating this cycle up to 18 times. In this manner, we could ensure that no loss of signal was obtained from the measurement of the sample mixture, while the measurement of the blank allowed us to ensure that no drifts on the baseline were being observed. After each cycle, cleaning of the electrode surfaces was carried out in phosphate buffer by recording a cyclic voltammogram under the same conditions. RSD% values of all the sensors were found to be below 4%, indicating good stability of the electrode responses. More specifically, the repeatability expressed as the percentage of RSD over 18 consecutive measurements of blank/standard cycles for the different electrodes forming the final array were: 2.4% for ZnO, 1.99% for PB, 2.4% for PPy and 3.6% for the Pt sensor.

### 3.3. Quantitative Analysis of APIs Mixtures

**Figure 5:**
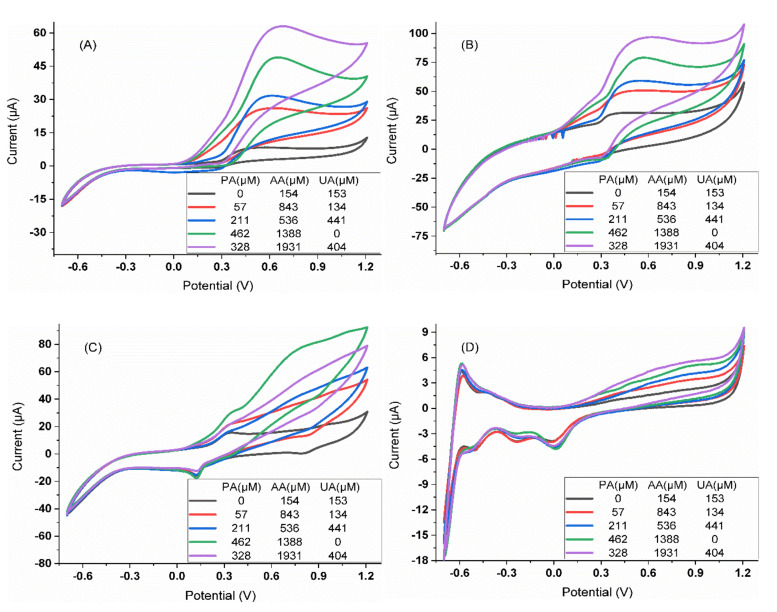
Representative voltammograms obtained for certain arbitrary mixtures of the different APIs (the concentration for each compound is indicated in the legend) with the four-sensor selected array: GECs modified with (**A**) ZnO, (**B**) PPy and (**C**) Prussian Blue, and (**D**) the metallic Pt electrode.

Upon selection of the optimal sensor array for the analysis of paracetamol, ascorbic acid, and uric acid, the next step was to evaluate the performance of such an array to achieve the simultaneous determination of their mixtures. To this aim, the set of samples described in Section 2.3 were measured under the same conditions as in previous experiments, recording a complete cyclic voltammogram for each of the electrodes (Figure 5). As could be expected from Figure 2, although a different voltammetric response is obtained for each of the considered compounds individually, there is a clear overlap when mixtures of those are analyzed simultaneously. Therefore, not being possible to achieve its quantification via univariate regression, requiring the aid of chemometric models; an approach that is possible thanks to the cross-response shown by the selected sensors, i.e., the different sensitivity shown by each of the electrodes towards each of the compounds (Table 1). This is a desirable condition for the proper performance of any multisensory array analysis system.

However, before building the quantification model, and especially if ANNs are to be used [32], a preprocessing step to reduce the high dimensionality of the data is required. The benefits derived from such a step are particularly critical with ANNs, as this contributes to preventing the under-determination problem encountered with an oversized ANN with excessively complex data, while it also reduces significantly the time and memory required for its modelling. Moreover, in general, it also leads to models with better performance and generalization ability as it avoids redundancy in the input data and reduces their complexity with the risk of overfitting [26]. In our case, this compression was achieved by means of DWT, using the Daubechies wavelet mother function and a fourth decomposition level. This allowed us to reduce the initial 1696 data points (currents corresponding to 424 polarization potentials × 4 sensors) down to 132 coefficients, representing a reduction of 92.2% without any loss of relevant information (r > 0.99 and fc > 0.95 in the compressed vs. original signal comparison).

For the selection of the neural network topology, a systematic study was carried out in which the number of neurons in the hidden layer, as well as the transfer functions between the input and hidden layer and between the hidden and output layers, were varied. The different ANN models were built employing the data of the training subset, and the selection of the optimal one was chosen from the comparison of the performance towards the testing subset. This data division helped to ensure that more unbiased data was obtained and to detect possible models that were being over-fitted, and consequently to better assess the accuracy of the model. The final ANN architecture had 132 neurons in the input layer (corresponding to the DWT coeffs., 33 × 4 sensors), 6 neurons and purelin transfer function in the hidden layer, and 3 neurons (one for each of the analytes) and satlins transfer function in the output layer.

**Figure 6:**
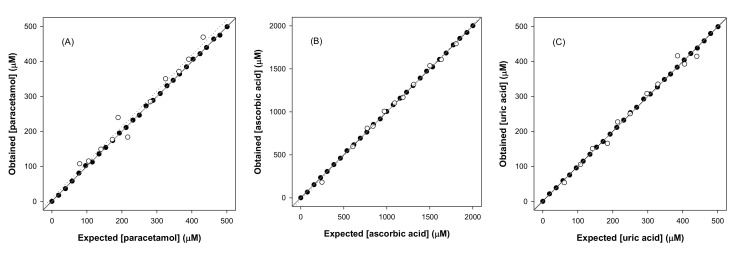
Modeling ability of the optimized DWT-ANN. Comparison graphs of obtained vs. expected concentrations for (**A**) paracetamol, (**B**) ascorbic acid, and (**C**) uric acid, for both the training (●, solid line) and testing subsets (○, dotted line). The dashed line corresponds to the ideal comparison line (y = x).

The comparison graphs of predicted vs. expected concentrations as well as the fitted linear regressions for the three determined species for the chosen ANN model are shown in Figure 6. As can be seen, a very satisfactory trend is obtained for all the cases, with regression lines almost overlapping or very close to the ideal ones. In order to better evaluate the goodness of the comparison, the regression parameters were also calculated and are summarized in Table 2. The obtained values are very close to the ideal values of slope (1), intercept (0), and correlation coefficient (1).

**Figure 7:**
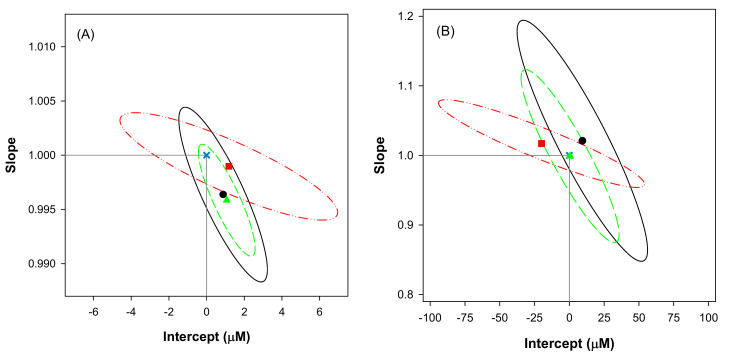
Joint confidence intervals for the three species: (●, solid line) paracetamol, (■, dash-dotted line) ascorbic acid, and (▲, dashed line) uric acid, and both for (**A**) training and (**B**) testing subsets. Also, the ideal point (1,0) is plotted (**x**); intervals are calculated at the 95% confidence level.

In this direction, to ensure that the former are statistically within the confidence intervals of the calculated regression parameters, the joint confidence intervals were calculated and plotted (Figure 7), as this allows to rapidly detect whether there are or not differences between the actual and predicted values at a certain significance level, judging simultaneously the goodness of slope and intercept [33]. In this case, the ideal point (1,0) is within the ellipsoidal confidence intervals for the three species, both for the training and testing subset, which allows us to state that there are no significant differences between the actual concentration and the values predicted by the model.

Despite the results for the training subset are more precise than those for the testing one, remarkable accuracy is obtained in both cases. The higher confidence intervals for the testing subset correspond to the usual behavior, which can be explained by two factors. On the one side, those samples are not used at all during the modelling stage, and consequently represent a more realistic metric of the model performance. On the other side, the lower number of samples of the testing subset in comparison to the training one results in higher tabulated *t* and F values that ultimately lead to higher confidence intervals. Similarly, the larger concentration range for ascorbic acid also results in a larger uncertainty for the intercept.

Finally, in order to benchmark the performance of the current proposed ET which is based on an optimized sensor array, its performance is compared to the one reported in previous works in which the same mixtures were analyzed (Table 2) [12,23,34]. To this aim, the root mean square error (RMSE) and its normalization (NRMSE) were also calculated to obtain a global metric of the system performance. Although some differences in the performance might be due to the different data treatment employed (e.g., PLS vs ANNs), it can be ruled out that a significant improvement has been achieved with the reduced sensor array. It can be seen that the smallest total NRMSE is obtained in the work reported here, but also, as could be expected from this, the improvement in the slopes and correlation coefficients is also highly significant.

## 4. Conclusions

The application of a simple methodology for the selection of the optimal voltammetric sensor array prior to carrying out a quantitative application has been demonstrated. The proposed approach is based on the combination of PCA/CVA to assess the cross-response of the different sensors with the F factor to numerically carry out the selection of the sensors from the scores plot.

To illustrate the potential of the methodology, the discrimination and quantification of three different APIs have been demonstrated. In this work, we initially selected an array of eight different electrodes, and from those, only four sensors were selected for the quantitative application. Next, the performance to carry out the quantitative determination of the three APIs was attempted by building a DWT-ANN model. The performance of the model was very satisfactory, and huge improvement was observed when benchmarked against other reported ETs attempting the quantification of the same mixtures. This confirms the potential advantages derived from the current approach, which allows the a priori selection of the potential best sensor array based on its cross-response features, the implicit reduction of the number of sensors (with the added advantage of instrumentation simplicity), and an improvement in the modelling performance (without requiring a posteriori pruning of the most relevant sensors).

Nevertheless, despite the good performance shown here, it has to be considered that there are many other clustering indexes, and that those are not universal. Therefore, future work has to focus on the comparison between different indexes and the suitability for different applications.

## Figures and Tables

**Table 1:** Calibration data (y vs. x) for the individual calibrations of paracetamol, ascorbic acid, and uric acid employing the final sensors in the array.

Electrode modifier		ZnO	Prussian Blue	Polypyrrole	Metallic Pt
Paracetamol	Equation	y = 0.0598x + 0.613	y = 0.0629x + 10.2	y = 0.0857x + 29.4	y = 0.0027x + 1.08
	R^2^	0.9971	0.9993	0.9989	0.9589
	LOD^1^ (μM)	28.4	13.9	17.2	59.4
Ascorbic acid	Equation	y = 0.0152x + 0.989	y = 0.0261x + 6.93	y = 0.0304x + 21.0	y = 0.0015x + 0.634
	R^2^	0.9977	0.9990	0.9987	0.9624
	LOD^1^ (μM)	59.3	68.5	77.5	417
Uric acid	Equation	y = 0.0452x + 1.12	y = 0.0499x + 10.8	y = 0.0668x + 31.0	y = 0.0028x + 1.02
	R^2^	0.9988	0.9983	0.9995	0.9967
	LOD^1^ (μM)	17.9	23.5	9.35	28.3

^1^ Calculated from three times the standard error of the regression.

**Table 2:** Reported values and current results of the fitted regression lines for the comparison between obtained vs. expected values for the different sets of samples and the three considered APIs. Intervals are calculated at the 95% confidence level.

Compound	Slope	Intercept (μM)	R^2^	RMSE^1^ (μM)	Total NRMSE^1^	Sensor Array	Ref.
training subset (n = 33)						Bare GEC plus metallic Pt and Au electrodes	[23]
Paracetamol	0.942 ± 0.031	32 ± 21	0.968	^2^	^2^		
Ascorbic acid	0.933 ± 0.040	36 ± 25	0.947	^2^			
Uric acid	0.873 ± 0.046	58 ± 25	0.923	^2^			
testing subset (n = 15)							
Paracetamol	0.895 ± 0.105	82 ± 71	0.848	^2^	^2^		
Ascorbic acid	0.919 ± 0.081	65 ± 41	0.908	^2^			
Uric acid	0.871 ± 0.138	−8 ± 86	0.753	^2^			
training subset (n = 33)						Bare GEC plus metallic Pt and Au electrodes	[34]
Paracetamol	0.981 ± 0.032	13 ± 24	0.992	29	0.0257		
Ascorbic acid	0.990 ± 0.031	6 ± 17	0.993	25			
Uric acid	0.981 ± 0.027	9 ± 16	0.994	23			
testing subset (n = 15)							
Paracetamol	0.990 ± 0.143	−2 ± 80	0.945	97	0.101		
Ascorbic acid	1.009 ± 0.136	−28 ± 78	0.952	66			
Uric acid	0.992 ± 0.208	36 ± 125	0.891	73			
training subset (n = 27)						SPCEs modified with CoPc, PB, graphite and CuO	[12]
Paracetamol	1.000 ± 0.082	0 ± 25	0.962	29	1.00		
Ascorbic acid	1.000 ± 0.089	0 ± 25	0.955	31			
Uric acid	1.000 ± 0.104	0 ± 31	0.940	36			
testing subset (n = 12)							
Paracetamol	1.021 ± 0.219	−13 ± 28	0.915	32	1.03		
Ascorbic acid	1.073 ± 0.422	−3 ± 54	0.762	71			
Uric acid	1.044 ± 0.334	−32 ± 36	0.829	44			
training subset (n = 27)						GECs modified with ZnO, PB, and PPy plus Pt metallic electrode	This work
Paracetamol	0.996 ± 0.006	0.9 ± 1.9	0.9998	2.43	0.00378		
Ascorbic acid	0.999 ± 0.004	1.1 ± 4.6	0.9999	5.86			
Uric acid	0.996 ± 0.004	1.1 ± 1.2	0.9999	1.64			
testing subset (n = 11)							
Paracetamol	1.021 ± 0.134	9 ± 36	0.971	26.4	0.0368		
Ascorbic acid	1.017 ± 0.049	−20 ± 57	0.996	31.2			
Uric acid	0.999 ± 0.096	1 ± 27	0.984	16.2			

^1^ RMSE: root mean square error; NRMSE: normalized root mean square error; ^2^ data not available; GEC: graphite epoxy composite; SPCE: screen printed carbon electrode.
